# Visualizing connectivity of ecological and evolutionary concepts—An exploration of research on plant species rarity

**DOI:** 10.1002/ece3.6633

**Published:** 2020-08-03

**Authors:** Thomas P. Wiegand, Braley Gentry, Zachary McCoy, Craig Tanis, Hope Klug, Michael B. Bonsall, Jennifer Nagel Boyd

**Affiliations:** ^1^ Department of Biology, Geology, and Environmental Science University of Tennessee at Chattanooga Chattanooga TN USA; ^2^ Department of Computer Science and Engineering University of Tennessee at Chattanooga Chattanooga TN USA; ^3^ SimCenter – Center for Excellence in Applied Computational Science and Engineering University of Tennessee at Chattanooga Chattanooga TN USA; ^4^ Department of Zoology University of Oxford Oxford UK

**Keywords:** network analysis, rare species, relative abundance, research gaps

## Abstract

Understanding the ecological and evolutionary factors that influence species rarity has important theoretical and applied implications, yet the reasons why some species are rare while others are common remain unresolved. As a novel exploration of scientific knowledge, we used network analysis conceptually to visualize the foci of a comprehensive base of >800 studies on plant species rarity within the context of ecology and evolution. In doing so, we highlight existing research strengths that could substantiate novel syntheses and gaps that could inspire new research. Our results reveal strong integrated foci on population dynamics with other ecological concepts. In contrast, despite the potential for ecological and evolutionary processes to interact, few studies explored the interplay of environmental factors and microevolutionary patterns. The cellular and molecular biology, physiology, and plasticity of rare plant species within both ecological and evolutionary contexts similarly provide avenues for impactful future investigations.

## 1 INTRODUCTION

Dramatic differences in the distribution and abundance of species in the natural world have been well documented historically (MacArthur, 1957; McGill et al., 2007; Preston, 1948; Rabinowitz, Cairns, & Dillon, 1986), and the question of why some species are rare while others are common has persisted (Darwin, 1859; Kruckeberg & Rabinowitz, 1985; May, 1999; McGill, 2006; Murray, Thrall, Gill, & Nicotra, 2002; Stebbins, 1942). Understanding such differences has vital implications for ecological and evolutionary theory as it relates to species relative abundance, as well as important applications to the conservation of species and overall biodiversity (Bevill & Louda, 1999). Rare species, in particular, comprise a disproportionate contribution to the ongoing extinction crisis (Van Calster et al., 2008), and although the ecological consequences of rare species loss often are underappreciated (Mouillet et al., 2013), rare species can act as biological indicators of overall habitat biodiversity (Lawler, White, Sifneos, & Master, 2003) and have been associated with various important roles in ecosystem functioning across a range of spatial and temporal scales (Mouillet et al., 2013), including roles in nutrient cycling (Theodose, Jaeger, Bowman, & Schardt, 1996), trophic food webs (Bracken & Low, 2012), and invasion resistance (Lyons & Schwartz, 2001; Zavaleta & Hulvey, 2004).

Species rarity is generally defined by low abundance, restricted range size, and/or habitat specificity (Rabinowitz et al., 1986), all of which can be impacted by a combination of ecological and evolutionary factors. While extrinsic environmental conditions such as climate, resource availability, and habitat stability can influence the occurrence and performance of species from an ecological perspective, such responses depend on intrinsic characteristics that are influenced by evolutionary processes such as genetic diversity and adaptive capacity (Sheth, Morueta‐Holme, & Angert, 2020). At the intersection of these disciplines, research that integrates focus on the ecology and evolution of rare species could lead to novel discoveries about the factors influential to species rarity (Pavoine & Bonsall, 2011). The rapid pace of contemporary environmental change due to anthropogenic activities and influences (Palumbi, 2001) has been implicated as a particular threat to rare species (Mouillet et al., 2013) and highlights the current relevance of research in this area. In particular, combined ecological and evolutionary research could elucidate potential acclimatory and adaptive constraints to species commonness within the context of such change.

At one extreme end of the continuum of species relative abundance, much attention has been given to advancing understanding of plant species invasiveness (Cadotte, Murray, & Lovett‐Doust, 2006; Daehler, 2003; Davidson, Jennions, & Nicotra, 2011; van Kleunen & Fischer, 2009; van Kleunen, Weber, & Fischer, 2010; Leffler, James, Monaco, & Sheley, 2014; Palacio‐López & Gianoli, 2011; Pyŝek & Richardson, 2007; Vanderhoeven et al., 2010). In contrast, research on plant species rarity—at the other end of the continuum—has been more limited (Combs, Lambert, & Reichard, 2013; Farnsworth, 2006; Murray et al., 2002). We aimed to characterize the comprehensive but relatively limited body of research on potential ecological and evolutionary drivers of plant species rarity to provide insight into research areas that have received substantial attention, as well as directions that warrant increased focus. To facilitate recognition of patterns in research foci (i.e., topics that have been studied in concert), we used network analysis, an approach to graph theory that symbolizes entities in a complex system using nodes connected by edges which can be weighted to show varying degrees of interaction or relation between individuals (Lesne, 2006). Traditionally, network analysis has been used by biologists to visualize gene and protein networks (Charitou, Bryan, & Lynn, 2016), food webs (Rakshit et al., 2017), plant–pollinator networks (Kovács‐Hostyánszki, Földesi, Báldi, Endrédi, & Jordán, 2019), and other community structures (Mello et al., 2019). Our less conventional use of network analysis to conceptualize published research on a topic was demonstrated previously in a characterization of research on invasive grasses, which illustrated that ecological concepts dominated that research relative to evolutionary concepts (Vanderhoeven et al., 2010). Our objective in using network analysis in this way was to highlight existing research strengths that could substantiate novel reviews and syntheses and research gaps that could inspire further research in the area of plant species rarity.

## 2 METHODS

### 2.1 Compiling a comprehensive literature base

We conducted an initial search in *ISI Web of Science* (Thompson Reuters) to screen the primary literature for studies of plant species in which their rarity or characterization as rare was a focus of the research. To accomplish this, we used the syntax *TS *= (plant* AND species) AND TI = (rar*) in the *ISI Web of Science* advanced search tool in March 2019 to return a list of 1,597 total articles, which we further filtered to those that were focused on vascular plants. We then personally screened the abstracts of the 1,499 remaining articles to identify studies that were empirical by eliminating reviews, proceedings, perspectives, and the like. Studies that were purely descriptive—those that catalogued rare species in an area, described rare species for identification purposes, and/or described specific locations housing rare species without offering broader insight into habitat requirements—were also eliminated along with those focused on taxonomy. In addition, we eliminated studies that focused solely on the recommendation or effectiveness of conservation measures without providing original scientific information about the species to help inform such measures, as well as articles that focused on the development and/or testing of methods that did not yield insights into species rarity (e.g., those that focused on rare species detection methods, ex situ propagation protocols, etc.). These steps yielded a resulting collection of 813 highly relevant studies of vascular plant species rarity to be characterized by our network analysis (File 1, Dryad, Dataset, https://doi.org/10.5061/dryad.bg79cnp8h). We acknowledge that a broader body of literature on species relative abundance could be applicable to understanding the factors that influence species rarity (as an extreme example of relative abundance), we wanted to highlight research explicitly focused on plant species rarity given the limited nature but important conservation applications of such research. Although it has been argued that knowledge of the biological characteristics of invasive species (as elucidated from a richer body of contemporary scientific knowledge) should be directly applicable to understanding species rarity as an opposite condition, tests of this concept have generated mixed results (25–29), suggesting that the applicability of studies of other manifestations of relative abundance to rarity is overly simplistic and that rare species merit distinct research attention.

### 2.2 Developing nodes and assigning keywords

The nodes in network analysis traditionally represent discrete but potentially interacting entities in a system—genes in an organism, individuals in a population, and species in a food web are biological examples. For the purpose of exploring between and across ecological and evolutionary research foci as interactions in a network, we aimed first to develop appropriate ecological and evolutionary nodes. In a previous use of network analysis to explore such integration in research of invasive grasses, which inspired our work, a discussion among experts at a workshop on the synthesis of ecology and evolution in invasive species studies resulted in a collection of nodes relevant to invasive species (Vanderhoeven et al., 2010). As a pilot project and to allow for direct comparison, we attempted to utilize these concepts to characterize a subset of the 813 articles that we deemed relevant to our exploration of research on species rarity. However, we encountered limitations in characterizing many of our articles according to their concepts (e.g., some common research foci did not relate well to any concepts while some concepts did not relate well to any research foci).

To capture more fully the research foci of our literature base and to facilitate comparable application to other study systems, we aimed to develop a network of nodes that represented ecology and evolution comprehensively as broad fields. This objective resulted in the development of 14 main “parent” nodes in three broad categories—Ecology, Evolution, and General Characteristics—that would allow for characterization of studies of plant species rarity as well as research in other potential study systems for which ecology and evolution are areas of interest (Table 1). Our Ecology category included nodes to describe environmental factors influential to and influenced by organisms, populations, species, and communities as well as a distinct node to reflect the subdiscipline of population ecology, which is a common focus of rare species studies. Evolution nodes focused on genetic diversity, mechanisms of evolution, and macroevolutionary patterns. The General Characteristics category included nodes descriptive of basic biological characteristics that could influence and be influenced by ecological and evolutionary factors but are not clearly ecological or evolutionary without context. To allow for more in‐depth examination of ecological and evolutionary research foci at a finer scale, we also developed lists of more specific “child” nodes for each of the major Ecology and Evolution Nodes (Table 1).

**Table 1:** Concepts/nodes and abbreviations used in our network analysis

Category	Parent Nodes	Child Nodes
Ecology	Abiotic Conditions	Climate Abiotic Disturbance Resources
	Anthropogenic Influences	Biological Invasions Climate Change Land Use Pollution
	Biotic Factors	Biotic Disturbance Competition Facilitation/Mutualism Herbivory Parasitism & Disease
	Population Dynamics	Demographic Factors Population Distribution Population Size
Evolution	Genetic Diversity & Systems	Heritability Ploidy Within Individual Variation Within Population Variation
	Macroevolution	Convergent Evolution Divergent Evolution Homologous Evolution* Hybridization Speciation
	Microevolution	Gene Flow Genetic Drift Mutation Natural Selection
General Characteristics	Cellular & Molecular Biology Growth & Development Physiology Niche Morphology Plasticity Reproduction	

* Node was conceptualized as a significant topic in the field of evolution; however, none of the KeyWords Plus from articles in our literature base were assigned to this node. As such, it was omitted from our networks.

The 813 relevant articles identified for our network analysis were added to a marked list in ISI Web of Science. We extracted the keywords from these articles from ISI Web of Science into a tab‐delimited file in Microsoft Excel (Microsoft). The resultant comprehensive list of keywords included both those assigned by the authors of the articles and the *KeyWords Plus*, which are generated by an algorithm in ISI Web of Science that performs derivative indexing wherein the titles of articles cited by the source text are searched for frequent terms that do not necessarily appear in the source text (Garfield & Sher, 1993). We chose to use *KeyWords Plus* for our network analysis because past research supports their use in lieu of author‐generated keywords as it eliminates personal bias by providing keywords that are not altered in any way by the author and illustrating a broader view of the literature base (Zhang et al., 2016). As many *KeyWords Plus* were assigned to multiple papers, we first removed duplicate cells from the file. The resultant list of *KeyWords Plus* was then filtered to remove those that were ambiguous or too broad for node assignment (e.g., “age,” “buffers,” “community,” “ecology,” “plant biology”), those that described types of rarity (e.g., “rare” and “endemic”), those that described methods, and keywords irrelevant to our research goals such as those that described specific species, plant types (e.g., “angiosperms,” “grasses,” “pines,” “trees”), specific locations, or habitat types (e.g., “desert,” “forest,” “tropical rainforest,” “tundra”). We also eliminated terms for which a search for one would return the others due to overlapping terminology (e.g., “life history traits” and “life history strategies” were eliminated because “life history” was a keyword). This filtering resulted in a final list of 486 total *KeyWords Plus* representing our comprehensive literature base. We then assigned each keyword to a parent node and child node (if applicable) based on general agreement among the authors (File 2, Dryad, Dataset, https://doi.org/10.5061/dryad.bg79cnp8h2).

### 2.3 Searching for concepts and building a network

Titles, keywords, and abstracts from our curated base of 813 articles focused on plant species rarity were extracted from the marked list assembled in ISI Web of Science to a text file. We generated a script in Perl, a programming language well suited for text mining (Bilisoly 2011), to identify the number of articles that focused on each pairwise combination of concepts based on a search of the title, abstract, author‐generated keywords, and *KeyWords Plus* of each article for the keywords that we assigned to each concept. The Perl script tagged each article with one or more of the concepts (Table 1) and counted the number of times each concept appeared in an article with every other concept.

We used diagonal matrices to organize data according to the total number of studies that were identified for each pairwise concept combination (Files 3, 4, Dryad, Dataset, https://doi.org/10.5061/dryad.bg79cnp8h). Connectivity values were then calculated as by Vanderhoeven et al. (2010) as the ratio of the number of studies that focused on a given pair of concepts to the total keywords relevant to those two concepts as a standard way to consider the relative influence of each concept combination (Files 3, 4, Dryad, Dataset, https://doi.org/10.5061/dryad.bg79cnp8h). The median of all connectivity values was calculated and the importance of each pairwise connection was considered relative to the median. Parent concepts involved in the five strongest pairwise connections were examined on a more detailed level by building a secondary network of the associated child nodes. Connectivity values below the median connectivity value were examined with a third network to highlight the least studied concept combinations.

We used Gephi visualization software, an open‐source platform for data visualization (Bastian, Heymann, & Jacomy, 2009), to visualize our concepts as nodes and connectivity values as weighted edges in a network. The 3‐D modeling capabilities of Gephi allowed us to generate networks representative of our literature base in a way that facilitated pattern recognition and the identification of strong and weak connections between concepts. Although Gephi outputs network visuals with nodes randomly arranged by default, layout algorithms can be used to arrange nodes in a meaningful way (Cherven, 2013). We applied the circular layout algorithm (Groeninger, 2013) available as a Gephi plugin to arrange the nodes in our network in a circular fashion, ordering concepts alphabetically within their categories for ease of interpretation. This layout algorithm is especially appropriate for networks such as ours that are relatively small (Cherven, 2013) and do not use force‐directed algorithms to represent interactions between entities in a physical system (Jacomy, Venturini, Heymann, & Bastian, 2014). In addition to investigating edge weight, which determined the influence that each pairwise combination of concepts had in the network, we considered the total number of edges connected to each node and their collected weights as a measure of the weighted degree of the importance of each concept in the network.

## 3 RESULTS

**FIGURE 1:**
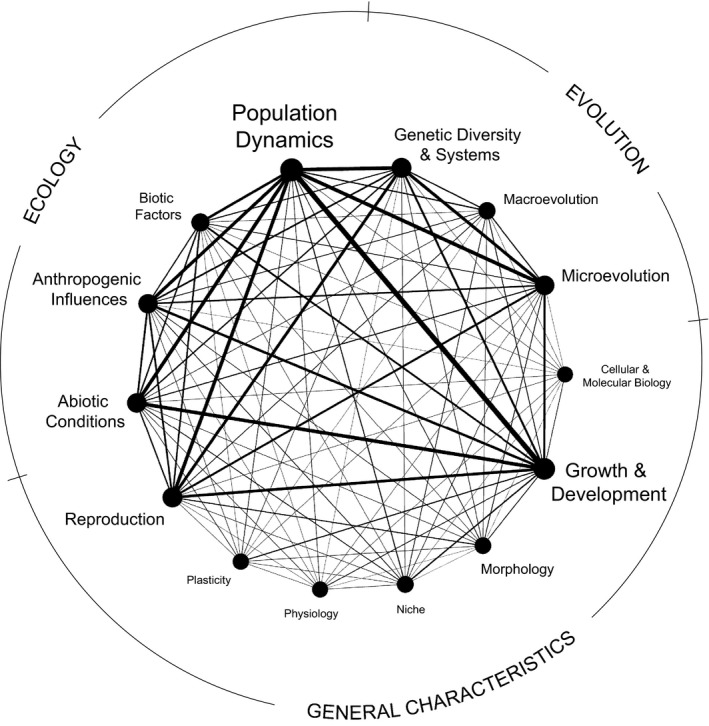
Network depicting connections and their strength between major (parent) ecological and evolutionary concepts and general plant characteristics as foci of published peer‐reviewed studies on plant species rarity. Nodes comprising the network perimeter represent ecology concepts (upper left area of the network), evolution concepts (upper right area), and general characteristics (bottom area) that were a focus of a comprehensive base of 813 research articles as determined from searches of associated keywords in the titles, abstracts, author‐generated keywords, and *Web of Science* Keywords Plus of the articles. The size of node labels is proportional to their overall focus in the collective literature base as determined by the average weighted degree of each node in our analysis. Line thickness is proportional to the number of studies connecting pairs of concepts standardized by the number of keywords representing each concept in the corresponding search

Our network analysis revealed that population dynamics was the most frequently studied concept across all studies of plant species rarity with an average weighted degree more than ten times greater than the least studied concepts (Figure 1; File 3, Dryad, Dataset, https://doi.org/10.5061/dryad.bg79cnp8h). There were especially strong connections between population dynamics and other ecological concepts in the literature base including abiotic conditions and anthropogenic influences, as well as with evolutionary concepts including genetic diversity and systems and microevolution. Population dynamics also exhibited strong connections with research on general plant characteristics including growth and development and reproduction. In contrast, our network revealed that other general plant characteristics were infrequently studied in combination with any ecological or evolutionary concepts; these included cellular and molecular biology, morphology, niche, physiology, and plasticity (Figure 1).

**FIGURE 2:**
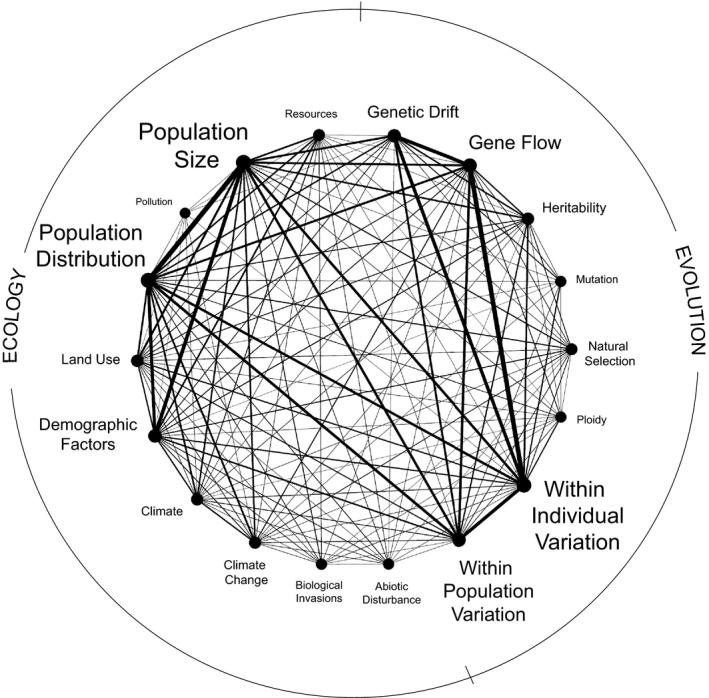
Network depicting connections between fine‐scale (child) nodes associated with the five most highly connected pairs of major (parent) ecology and evolution concepts (see Figure 1) and their strength as foci of published peer‐reviewed studies on plant species rarity. Nodes comprising the network perimeter represent ecology concepts (left side of the network), and evolution concepts (right side) that were a focus of a comprehensive base of 813 research articles as determined from searches of associated keywords in the titles, abstracts, author‐generated keywords, and *Web of Science* Keywords Plus of the articles. The size of node labels is proportional to their overall focus in the collective literature base as determined by the average weighted degree of each node in our analysis. Line thickness is proportional to the number of studies connecting pairs of concepts standardized by the number of keywords representing each concept in the corresponding search

Overall, all of the five most connected concept pairs in our main network involved population dynamics, with the exception of a relatively strong connection between genetic diversity and systems and microevolution. Investigation of child nodes associated with concepts in the five most connected pairs revealed that all aspects of population dynamics (i.e., distribution, size, and demography) were well studied (Figure 2; File 4, Dryad, Dataset, https://doi.org/10.5061/dryad.bg79cnp8h). At the intersection of ecology and evolution, a relatively large number of studies focused on relationships between population dynamics and microevolutionary forces with particular emphasis on gene flow and genetic drift. Individual‐ and population‐level genetic variation were also well studied in the context of population dynamics. Within ecology, population dynamics were often studied in combination with research foci on climate factors, land use, and resource availability. Although anthropogenic influences were relatively well represented overall as a parent node in our main network, studies connecting biological invasions and/or pollution to rarity were limited (Figure 2).

**FIGURE 3:**
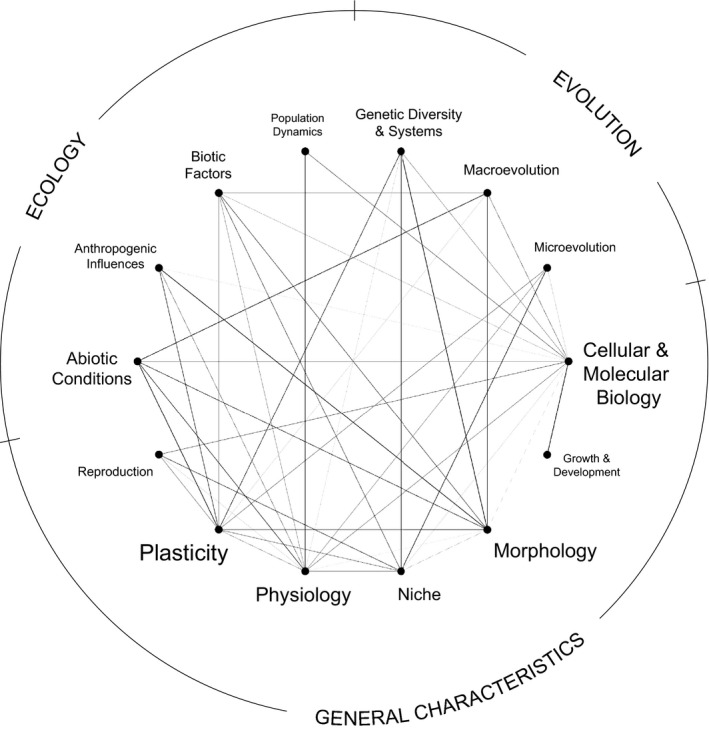
Network depicting connections between major (parent) ecology and evolution concepts (see Figure 1) below the median connectivity value (i.e., relatively weak connections) and their strength as foci of published peer‐reviewed studies on plant species rarity. Nodes comprising the network perimeter represent ecological concepts (upper left area of the network), evolutionary concepts (upper right area), and general characteristics (bottom area) that were a focus of a comprehensive base of 813 research articles as determined from searches of associated keywords in the titles, abstracts, author‐generated keywords, and *Web of Science* Keywords Plus of the articles. The size of node labels is proportional to their overall focus in the collective literature base as determined by the average weighted degree of each node in our analysis. Line thickness is proportional to the number of studies connecting pairs of concepts standardized by the number of keywords representing each concept in the corresponding search

Our network highlighting weak connections (i.e., those with connectivity values below the median value) and connectivity gaps revealed that many general characteristics of rare species have not been well studied in an ecological and/or evolutionary context, especially cellular and molecular biology, physiology, and plasticity (Figure 3; File 3, Dryad, Dataset, https://doi.org/10.5061/dryad.bg79cnp8h). Although such connections were limited overall, the only relevant network gap revealed was between physiology and macroevolution. Because no articles reported research on homologous evolution in combination with any other concept in our analysis—including general plant characteristics and ecological and evolutionary concepts—homologous evolution was not integrated into a network (Figure 3); however, this topic does represent another gap in the research base.

## 4 DISCUSSION

### 4.1 Opportunities for reviews and syntheses

The strong focus on population dynamics in studies of plant species rarity (Figure 1), including both population size and distribution (Figure 2), is not surprising given the conservation applications of understanding population trends for rare species, which generally have high extinction probabilities and thus can contribute disproportionately to overall biodiversity loss (Mouillet et al., 2013; Myers, Mittermeier, Mittermeier, da Fonseca, & Kent, 2000). Heavily connected links between population size and/or distribution and other ecological and evolutionary concepts in our main network (Figure 1) reveal opportunities for reviews and syntheses, such as potential meta‐analyses, that could increase our understanding of the extrinsic and intrinsic influences of rarity. For example, studies that integrate research foci on population dynamics with other ecological concepts could provide insight into environmental factors that influence such dynamics. The rapid pace of contemporary environmental change due to human activities has been implicated as a particular threat to rare plant species (Mouillet et al., 2013), and we suggest that an ecological review of relationships between anthropogenic and abiotic influences and the population dynamics of rare plant species is likely possible given the strong connections between these concepts in the literature base (Figure 1), especially within the context of the relative influences of climate change and land use (Figure 2).

Several previous syntheses returned by our initial *ISI Web of Science* search investigated links between population size and biotic interactions including insect herbivory, pollinator activity, and host–parasite relationships within the context of plant species rarity. The most recent of these syntheses was an investigation of the influence of insect herbivory on 35 rare and endangered plants and more than 60 insect species found impacts that were largely negative, but reported that a few studies suggested that insect herbivores can positively impact rare species by stabilizing population numbers in ways that minimize density‐dependent negative factors (Ancheta & Heard, 2011). In a previous discussion of plant–pollinator interactions supported by published research, Spira (2001) reported that small and low‐density plant populations, as are characteristic of many rare species, could be negatively impacted by reduced pollinator activity as the result of declines in native pollinator populations. An earlier review focused on rare parasitic plant species, in particular, found that the host requirements of rare parasitic plants were not well known although these requirements would be important to consider for effective rare species conservation (Marvier & Smith, 1997). Our search returned only one synthetic report that focused primarily on links between population ecology and anthropogenic influences within the context of plant species rarity. Specifically, a meta‐analysis revealed that populations of rare plant species were susceptible to reduced genetic diversity as a consequence of habitat fragmentation, but that common species were equally susceptible to such impacts (Honnay & Jacquemyn, 2007).

Studies connecting population dynamics and evolutionary concepts could provide mechanistic understanding of how adaptive and nonadaptive processes drive the population dynamics of rare species. Our network reveals that a relatively large body of published research has integrated ecological focus on the population dynamics of rare species with microevolution (Figure 1), suggesting that reviews of the relative importance of natural selection, genetic drift, gene flow, and mutation on the population size and distribution of rare plant species are likely warranted (Figure 2). Our *ISI Web of Science* search did not return any existing reviews in this area, further reinforcing this need. Although our search results did include a review of the influence of hybridization on rare species loss via demographic swamping and genetic assimilation (Levin, Francisco‐Ortega, & Jansen, 1996), our network analysis revealed that links between macroevolutionary concepts and population dynamics were not particularly well studied (Figure 1). Our search also found an extensive meta‐analysis of 95 rare and more than 150 common plant taxa that revealed that genetic diversity may be reduced in rare plant species compared with common plant species (Cole, 2003). A broader review of connections between individual‐ and population‐level genetic diversity and population dynamics of rare plant species could help to elucidate the evolutionary causes and consequences of such diversity. Overall, the syntheses that we gathered provide insight into a variety of ecological and evolutionary factors potentially influential to species rarity, but such reports are notably limited in number and do not include the past decade of relevant research. As such, the results of our network analysis should encourage future synthetic investigations of both ecological and evolutionary aspects of rarity.

### 4.2 Avenues for further research

In contrast to well‐studied foci that could be subject to robust review, weakly connected concepts in our network suggest opportunities for further research that could advance understanding of rarity. For example, despite the potential for ecological and evolutionary processes to interact in ways that could influence species success (Vanderhoeven et al., 2010), few studies have considered the interplay of the abiotic, biotic, or anthropogenic environment on microevolutionary patterns in rare plant species (Figure 1). However, environmental factors such as climate, competition, and habitat modification, all of which can be influenced by human activities, are well known to have both independent and interactive effects on plant fitness and population dynamics across life forms, latitudes, and biomes (Morris, Ehrlén, Dahlgren, Loomis, & Louthan, 2020). At a broader scale, limited research has explored connections between ecological and macroevolutionary concepts (Figure 3) within the context of rarity although ecological factors can influence the occurrence, direction, and rate of trait evolution, speciation, and extinction (Davies, 2019; Weber, Wagner, Best, Harmon, & Matthews, 2017). Given these gaps, future research with focus on links between the environment and microevolutionary and macroevolutionary patterns in rare species have potential to advance our understanding of rarity. Indeed, there is evidence that extinction risk is distributed nonrandomly across space and phylogeny, and there has been a call for enhanced understanding of the link between macroevolutionary patterns and the interaction between ecological factors, selective pressures, and the evolutionary history of species (Davies, 2019; Weber et al., 2017). In particular, exploring how evolutionary history, ecology, and selection interact to influence the geographic distribution and extinction rates of rare plants will be a particularly important focus of future research.

The general lack of research connecting physiology and cellular and molecular characteristics of rare plant species with their ecology and evolution (Figures 1 and 3) suggests that intrinsic nongenetic mechanisms potentially influential to rarity also are poorly understood. Although dramatic differences in ecophysiological traits such as gas‐exchange activity, tissue biochemistry, and resource‐use efficiency that underlie variation in individual growth and fitness and ultimately population‐ and community‐level performance have been evidenced between species (Ackerly et al., 2000; van Kleunen et al., 2010), such differences have not been well investigated within the context of species rarity, which limits our understanding of the potential ability of rare species to adapt to changing environmental conditions. In addition, relatively little is known about how environmental and subsequent physiological changes influence the cellular environment, including cellular temperature, pH, and acid–base balance, and few studies have examined how such molecular changes influence ecology and evolution in general (Seebacher & Franklin, 2012). Some authors have suggested that focusing on such physiological, cellular, and molecular mechanistic responses to environmental change is key for understanding ecological and evolutionary patterns (Seebacher & Franklin, 2012). In contrast, the inclusion of physiological traits in studies comparing noninvasive and invasive plant species—at the opposite extreme end of species relative abundance—has been relatively common (Van Kleunen et al., 2010) with such traits being found to differ significantly between these groups, suggesting the potential for similar application of physiology to studies of species rarity.

A further understudied area in the study of plant species rarity is the role that plasticity may play within the context of rarity in relation to both ecology and evolution (Figures 1 and 3). This research limitation is perhaps surprising because plasticity could influence organismal fitness, natural selection, and ultimately species performance and adaptation in ways that could facilitate expansion across varied environments and/or persistence in locations experiencing environmental change (Godoy, Valladares, & Castro‐Díez, 2012; Nicotra & Davidson, 2010). As sessile organisms, plants are characterized by relatively high degrees of trait plasticity (Sultan, 2000) and plant species and populations can differ extensively in their responses to environmental change due to plasticity differences (Valladares, Gianoli, & Gómez, 2007), which could help to explain the constrained geographic distribution of many rare species. Studies examining the uniformity/diversity of how closely related species varying in habitat specificity also have suggested a link between plasticity and rarity as it could have implications for the persistence and dynamics of habitat specialists (Murray et al., 2002). Although a review published nearly two decades ago highlighted the lack of explicit research on plasticity as potentially influential and consequential to plant species rarity (Murray et al., 2002), investigations of plasticity in this context have remained limited, generally consisting of studies focused on single genera that collectively produced mixed findings (Denton, Venklaas, & Lambers, 2007; Lovell & McKay, 2015; Marchin et al., 2009; Pohlman, Nicotra, & Murray, 2005; Rünk & Zobel, 2007). However, such studies have suggested that considering the plasticity of physiological traits in particular (Pohlman et al., 2005) and combining investigations of trait plasticity and genetic diversity (Lovell & McKay, 2015), a concept that has been well studied within the concept of species rarity (Figure 1), are likely to be promising avenues for further exploration. Within the context of biological invasions, researchers have reported positive associations between invasiveness—at the extreme opposite end of the relative abundance spectrum—and plasticity (Ruprecht, Fenesi, & Nijs, 2014), particularly when physiological traits were considered (Davidson et al., 2011; Funk, 2008; Godoy et al., 2012), further demonstrating the potentially important role that plasticity of such traits could play in species relative abundance.

The understudied areas of research revealed by our network analyses could be the result of logistical limitations that prevent researchers from examining such links. Inherently, research of rare species can be impeded by overall limited plant availability and difficulty in locating and individuals for robust investigations of organismal‐level properties such as physiological processes and cellular and molecular characteristics. However, such investigations are possible. For example, Baskauf and Eickmeier (1994) examined the link between photosynthetic performance in relation to light and soil moisture preconditioning regimes in a rare and common *Echinacea* species. Microevolutionary and plasticity studies on rare plants have utilized controlled laboratory or field conditions to quantify fitness metrics in relation to abiotic or biotic ecological factors (see Lammi, Siikamäki, & Mustajärvi, 1999; Sætersdal & Birks, 1997; Scarano, 2009). While large and comprehensive abiotic datasets that could be used in broader syntheses exist (e.g., long‐term climate data is readily available through NOAA and NASA), biotic data on rare plants are in many cases not readily available. For example, the PLANTS Database (USDA & NRCS, 2020) provides taxonomic and habitat information for a range of plant species, but there is relatively less information on rare plants. Although NatureServe Explorer (NatureServe, 2020) provides ecological and life history information for many plant species, including those that are rare and of conservation interest, information about physiology and plasticity that could be assessed in conjunction with available abiotic data is limited.

In addition to more data collection, it is possible that a more robust conceptual framework that incorporates the understudied links is needed. Specifically, it would be beneficial for future theoretical research to identify clear hypotheses and predictions related to ecology, microevolutionary and macroevolutionary patterns, physiology and cellular molecular characteristics, and plasticity. For example, species distribution models have previously been used to generate predictions of habitat suitability of rare plants (Gogol‐Prokurat, 2011). Such models could potentially be used to generate hypotheses and predictions of how abiotic and biotic factors, individual characteristics, and plasticity could influence micro‐ and macroevolutionary patterns, including species distributions and rates of extinction. Such hypotheses and predictions might then provide a conceptual framework that could better guide and motivate research on these understudied links.

## 5 CONCLUSIONS

Our application of network analysis to a comprehensive collection of research focused on plant species rarity allows for characterization of this body of scientific knowledge in a visual way that readily reveals its strengths and weaknesses. In combination, syntheses of research with well‐studied foci (i.e., strengths) and empirical investigations focused on research “gaps” (i.e., weaknesses) as informed by our network analysis could help to advance ecological theory about species rarity and guide the conservation of rare species and overall biodiversity as a result.

Our use of network analysis to characterize research foci expands upon its previous use in this way (see Vanderhoeven et al., 2010) by utilizing a hierarchical consideration of concepts from broad parent nodes to more specific child nodes that allow for a finer‐scale research characterization. The use of text mining software to extract relevant keywords allowed for efficient, simultaneous searching of a collection of studies. Following research characterization, further network analysis could be used to synthesize the findings of articles sharing strong concept connections by defining treatment levels and research outcomes as nodes in a network (Vanderhoeven et al., 2010). Although we constrained our network analysis to include studies that explicitly focused on plant species rarity, we acknowledge the potential for research on various characterizations of relative abundance—geographical distribution, habitat specificity, local abundance (see Rabinowitz et al., 1986)—to be potentially applicable to understanding rarity and suggest that future syntheses consider such research. More broadly, we suggest the utility of network analysis—as applied to the characterization of research on plant species rarity here—as an effective way to explore research in other areas toward elucidating strengths for reviews and gaps to guide future investigations and to synthesize findings in a visual way.

## CONFLICT OF INTEREST

The authors declare no competing financial interests.

## AUTHOR CONTRIBUTIONS

**Thomas P. Wiegand:** Formal analysis (lead); writing–original draft (equal). **Braley Gentry:** Formal analysis (supporting). **Zachary McCoy:** Methodology (supporting). **Craig Tanis:** Methodology (lead); writing–review and editing (equal). **Hope Klug:** Data curation (supporting); funding acquisition (equal); writing–original draft (equal). **Michael B. Bonsall:** Conceptualization (supporting); formal analysis (supporting); writing–review and editing (equal). **Jennifer Nagel Boyd:** Conceptualization (lead); data curation (lead); funding acquisition (equal); writing–original draft (equal).

### Open Research Badges

This article has earned an Open Data Badge for making publicly available the digitally‐shareable data necessary to reproduce the reported results. The data is available at https://doi.org/10.5061/dryad.bg79cnp8h.

## Data Availability

Bibliographic information for comprehensive literature base, parent and child nodes/concepts and article keyword assignments, and matrices showing connectivity values of pairwise combinations of parent and child nodes/concepts: Dryad, Dataset, https://doi.org/10.5061/dryad.bg79cnp8h
